# Novel Osteointegrative Sr-Substituted Apatitic Cements Enriched with Alginate

**DOI:** 10.3390/ma9090763

**Published:** 2016-09-08

**Authors:** Simone Sprio, Massimiliano Dapporto, Monica Montesi, Silvia Panseri, Wanda Lattanzi, Enrico Pola, Giandomenico Logroscino, Anna Tampieri

**Affiliations:** 1 Institute of Science and Technology for Ceramics, National Research Council of Italy, Via Granarolo 64, Faenza 48018, Italy; massimiliano.dapporto@istec.cnr.it (M.D.); monica.montesi@istec.cnr.it (M.M.); silvia.panseri@istec.cnr.it (S.P.); anna.tampieri@istec.cnr.it (A.T.); 2 Institute of Anatomy and Cell Biology, Università Cattolica del Sacro Cuore, Largo F Vito, 1, Rome 00168, Italy; wanda.lattanzi@unicatt.it; 3 Latium Musculoskeletal Tissue Bank, Largo F Vito, 1, Rome 00168, Italy; 4 Orthopaedics and Traumatology, Università Cattolica del Sacro Cuore, Largo F Vito, 1, Rome 00168, Italy; enricopola@hotmail.com (E.P.); g.logroscino@fastwebnet.it (G.L.)

**Keywords:** bone cement, hydroxyapatite, strontium, alginate, in vivo, osteointegration

## Abstract

The present work describes the synthesis of novel injectable, self-setting bone cements made of strontium-substituted hydroxyapatite (Sr-HA), obtained by single-phase calcium phosphate precursors doped with different amounts of strontium and enriched with alginate. The addition of alginate improved the injectability, cohesion, and compression strength of the cements, without affecting the hardening process. A Sr-HA cement exhibiting adequate hardening times and mechanical strength for clinical applications was further tested in vivo in a rabbit model, in comparison with a commercial calcium phosphate cement, revealing the maintenance of biomimetic composition and porous microstructure even after one month in vivo, as well as enhanced ability to induce new bone formation and penetration.

## 1. Introduction

The development of injectable and self-hardening biomaterials able to regenerate damaged bones by minimal invasive surgery is a topic of increasing interest, particularly for the treatment of vertebral fractures [1,2,3,4,5], but also tibial plateau, proximal humerus, wrist and calcaneus [6], as well as hip and femoral neck [7].

In this respect, complete bone regeneration still remains an unsolved clinical need, mainly due to the unavailability of bone cements endowed with adequate bioactivity, porosity, and osteoconductivity able to promote effective new bone formation and extensive cell proliferation upon hardening. Indeed, it has been reported that the main drawback exhibited by commercially available bone cements is the inability to be completely colonized by the new bone, thus affecting the establishment of well-integrated bone-biomaterial constructs [8,9,10]. The impairment of the mechanical performance may be due to both the excessive stiffness of the cement, as reported in the case of acrylic formulations [11,12,13,14], and the low porosity, as reported in the case of calcium phosphate bone cements (CPCs) [1,15].

The hardening of acrylic bone cements (e.g., poly(methylmethacrylate), PMMA) is also reported to occur through an exothermic polymerization, leading to the necrosis of the surrounding tissues [16]. In this respect, CPCs are considered as elective candidates for bone regeneration due to their excellent biocompatibility, chemical similarity with bone tissue, and ability to harden in vivo at body temperature. 

Several approaches have been proposed to prepare CPCs; among them, the one based on the hydrolysis and transformation of *α*-Ca_3_(PO_4_)_2_ (*α*TCP) into elongated calcium-deficient hydroxyapatite (HA) particles is particularly interesting and promising [1]. Indeed, the chemical reactions involved in the cement hardening are isothermal and not subjected to pH variations, thus avoiding potential cell injury. Calcium-deficient HA is also characterized by increased bioactivity and bio-resorbability, if compared with stoichiometric HA [17]. Moreover, such cements can be endowed with enhanced bioactivity by introduction of bio-competent ions (e.g., Mg^2+^, CO_3_^2−^, SiO_4_^4−^, and Sr^2+^) in the HA lattice as substitutes of calcium or phosphate [18,19,20,21]. Strontium has been largely studied due to its proven ability to restore the bone turnover balance, especially when the treatment of bone fractures caused by osteoporosis is demanded [22,23]. As systemic pharmacological approaches based on Sr-containing drugs often resulted in deleterious effects on bone mineralization due to reduction in calcium absorption and possible alterations of the properties of the mineral bone [24], a controlled delivery of relevant active principles in situ is highly desirable to provide safe and efficient therapies against degenerative bone diseases. Some positive results on the efficacy of local release of strontium were obtained in recent studies based on titanium implants functionalized with strontium [25] or coated with Sr-substituted HA [26]. In addition, Sr-containing CPCs based on commercial *α*TCP and soluble salts containing strontium (e.g., SrCO_3_, SrHPO_4_) were previously developed [27,28,29] and tested in ovariectomized rats [30]. 

A major challenge for material scientists is the control of the chemistry and rheological properties of injectable CPCs; indeed, process parameters, such as phase composition of the inorganic precursor, particle size, liquid-on-powder ratio (L/P), polymeric additives are critical factors affecting their viscosity, cohesion, and setting times [15]. In this respect, previous research reported improved cement injectability and cohesion by decreasing the particle size of the starting powders, as well as using more viscous solutions. However, in such conditions the increase of the cement cohesion was also associated to an impairment of setting times and mechanical performance [31]. It was also reported that smaller precursor particles favor the transformation into apatite, as well as the establishment of a more packed structure and higher mechanical strength [32,33,34]. On the other hand, the introduction of foreign ions into the calcium phosphate structure prolonged the setting times [29,35]. Among the various approaches proposed to improve the hydraulic reactivity of the precursor powders, the high-energy planetary ball milling was reported to be particularly promising in terms of optimal particle size reduction efficiency [36]. Previous research was also dedicated to improve the rheological properties of the cement by addition of organic compounds such as biopolymers [15]. In this respect, alginate was reported as a biomaterial for bone tissue engineering, due to its biocompatibility, non-toxicity, and biodegradability, as well as for its strengthening ability [37]. 

In the present work Sr-doped HA cements were prepared by mixing Sr-substituted *α*TCP phases, appositely designed and synthesized to be the unique inorganic precursors, with disodium phosphate solutions enriched with alginate. The process parameters, e.g., the powder milling and liquid-to-solid ratio, were optimized to obtain adequate setting times and mechanical properties. The in vivo performance of a selected cement formulation was preliminary evaluated at four weeks after surgery into rabbit femur, in comparison with a commercial CPC, by compositional, morphological, and histological/histomorphometric analysis.

## 2. Results

### 2.1. Physicochemical Properties and Setting Behavior

The ICP analysis of the Sr-*α*TCP phases detected the presence of strontium in amounts very close to the nominal composition of the starting mixture (see Table 1). 

**Figure 1:**
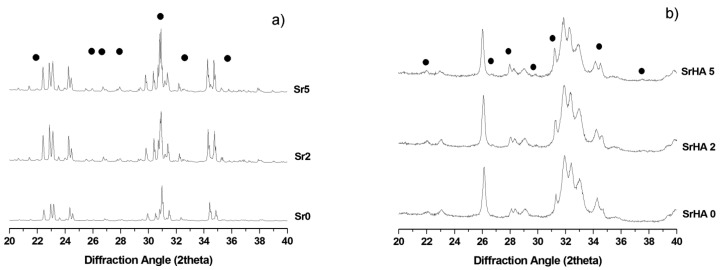
XRD diffraction profiles of: (**a**) cement precursors with different Sr content (Sr/Ca: 0–5 mol %); (**b**) the corresponding hardened cements after 3 days of incubation at 37 °C. The • symbol indicates the peaks belonging to *β*TCP phase. In Figure 1a the non-indexed peaks address *α*TCP phase, whereas in Figure 1b, the HA phase.

The XRD patterns of the as-obtained powders show peaks that can be ascribed to *α*TCP (ICDD: 29-0359) and *β*TCP phases (ICDD: 09-0169). No XRD reflections belonging to other crystalline phases were detected (see Figure 1a). Semi-quantitative evaluation of the phase composition reported the formation of *β*TCP phase (in the range 7–12 wt %) in the as-prepared materials, slightly increasing with the strontium content. 

The phase composition of the cements obtained after three days of incubation at 37 °C in a thermostated water bath was also investigated, showing the complete transformation of *α*TCP into HA phases, while preserving small amounts of *β*TCP (see Figure 1b).

A steady shift of the peaks towards higher *d* values and a quasi-linear increase of the lattice parameters were observed in both *α* and *β*TCP phases. A similar effect was also detected for the HA phases of the corresponding hardened cements (Table 2).

The Sr-free *α*TCP phase (Sr0) exhibited a domain size *D* = 362 ± 8 nm and a steady increase of the coherent domain was observed by increasing the strontium content, according to the quasi-linear trend *D* = 299.6 + 11.4*x* (R^2^ = 0.98), where *x* is the Sr/(Ca + Sr) percentage molar ratio. The domain size of *β*TCP showed much lower values, in the range 100–150 nm. 

**Figure 2:**
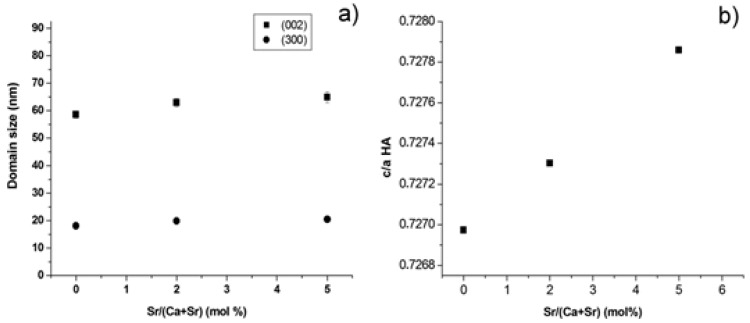
(**a**) Coherent domain size in Sr-HA phases along the *ab* plane and the *c* axis; and (**b**) distortion of the HA lattice with strontium doping.

The average domain size of Sr-HA was about 25 nm, nearly irrespective of the strontium amount. The full width half maximum of (300) and (002) reflections (*d* ≌ 2.72 and 3.44 Å, respectively) were also separately evaluated, thus achieving the size of coherent domains along both the *ab* plane and the *c* axis: it was found that the introduction of strontium progressively induced anisotropic crystal growth along the *c* axis (Figure 2a), leading to the distortion of the HA lattice (Figure 2b) according to the quasi-linear trend *c*/*a* = 0.73 + 1.8 × 10^−4^*x* (R^2^ = 0.99), where *x* is the Sr/(Ca + Sr) percentage molar ratio.

**Figure 3:**
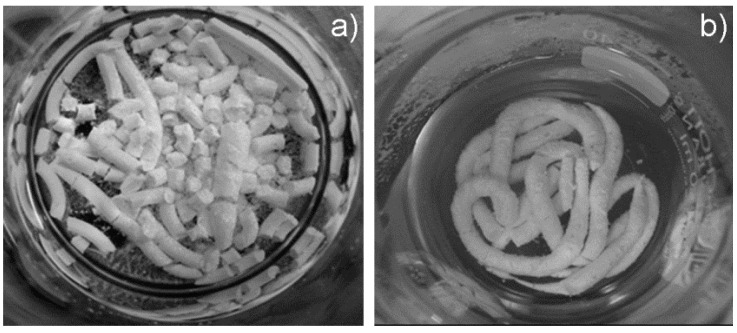
Cohesion of the cements after 30 min of immersion in Hanks’ balanced salt solution at 37 °C. (**a**) Sr5 without alginate; and (**b**) Sr5 with alginate.

The cement formulations enriched with alginate exhibited improved injectability and cohesion of the paste, without demixing or cement fragmentation after 30 min of immersion into Hanks’ balanced salt solution at 37 °C (Figure 3).

**Figure 4:**
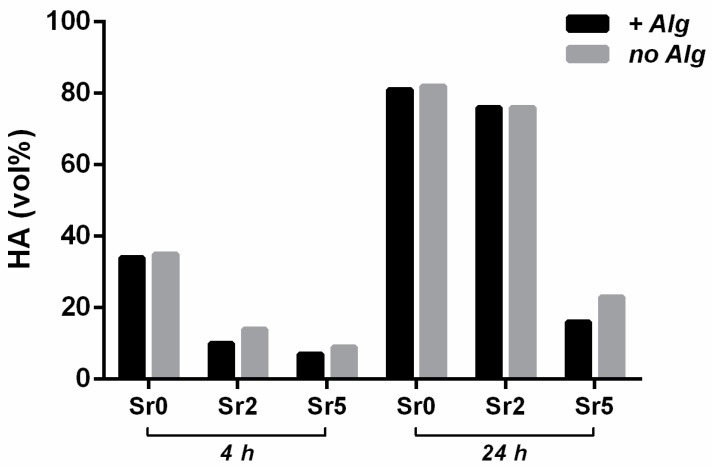
Progress of the transformation of *α*TCP precursors into HA over time, with and without alginate.

The presence of alginate did not negatively affect the setting reactions, while a weak retarding effect on the phase transformation was observed, especially for Sr5. On the other hand, the introduction of strontium retarded both the setting times and the transformation of Sr-*α*TCP into Sr-HA (Table 3 and Figure 4).

### 2.2. Morphological and Mechanical Aspects

**Figure 5:**
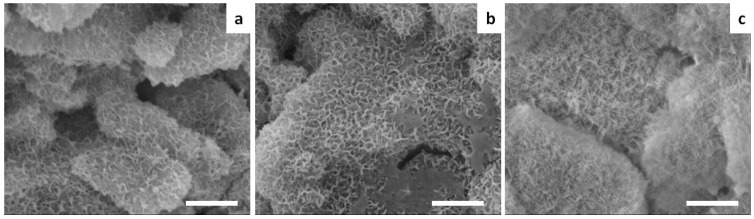
SEM micrographs of (**a**) Sr0; (**b**) Sr2; and (**c**) Sr5 cements. Scale bar: 2 µm.

The Sr-doped cements exhibited nano-sized elongated crystals developed upon heterogeneous nucleation and growth on the surface of the precursor particles (Figure 5), thus enabling the establishment of an extensive micron-sized porosity, irrespective of the extent of strontium doping.

The overall porosity of Sr0, Sr2 and Sr5 cements was calculated as 51% ± 2%, 46% ± 3%, and 49% ± 1%, respectively. 

**Figure 6:**
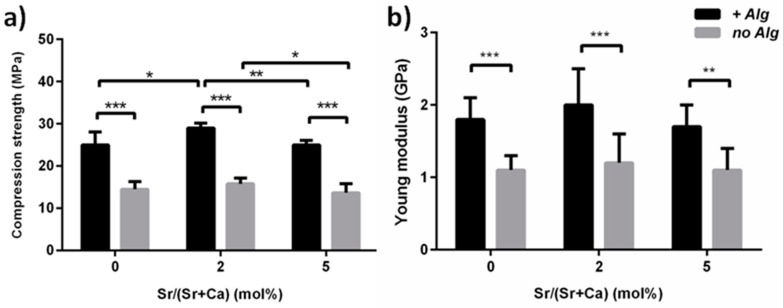
Effect of alginate on the compressive strength (**a**) and Young’s modulus (**b**) of Sr-doped cements after three days of immersion. * *p* < 0.5; ** *p* < 0.1; *** *p* < 0.01.

The presence of alginate significantly improved the compressive strength and Young’s modulus of the cements at three days after immersion into Hanks’ balanced salt solution at 37 °C, when compared with alginate-free formulations (Figure 6). 

The Sr2 cement exhibited the highest compressive strength. The long-term compressive strength of Sr2 was also tested, exhibiting *σ* = 28.1 ± 1.2 MPa and Young’s modulus E = 2.3 ± 0.2 GPa after 30 days of immersion in physiological conditions. 

**Figure 7:**
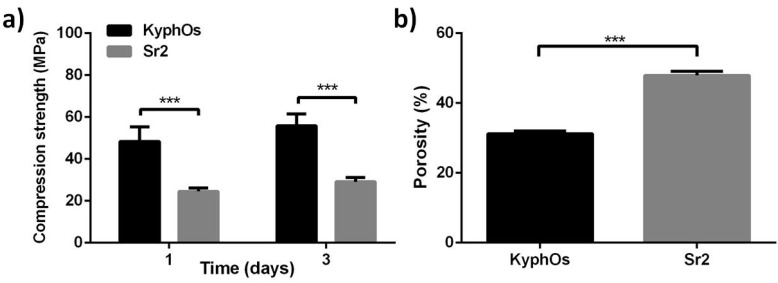
Evaluation of Sr2 cement, compared with control cement: compressive strength (**a**) and porosity after three days of incubation (**b**). *** *p* < 0.01.

On the basis of these results, the compressive strength of Sr2 cement was also tested in comparison with KyphOs cement, which was the control of the in vivo tests. At one, and after three, days of incubation, the KyphOs cement exhibited higher compressive strength than Sr2, and also significantly lower porosity (Figure 7).

### 2.3. Biological Evaluation by in Vitro Cell Tests

**Figure 8:**
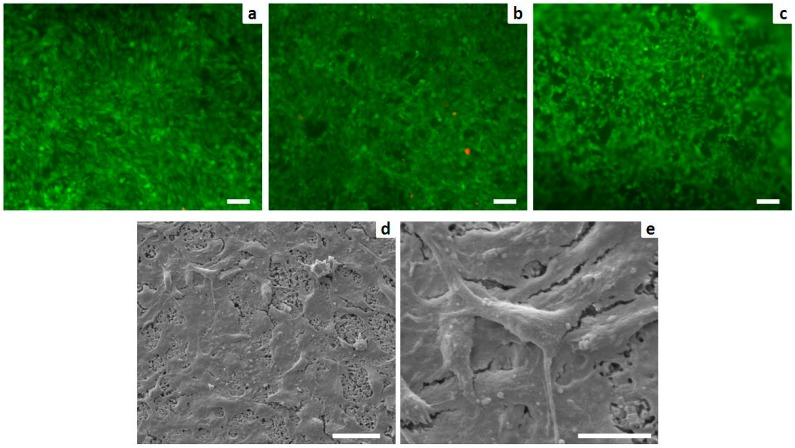
Cell viability analyzed by the live/dead assay: Calcein AM stains for live cells in green, EthD-1 stains for dead cells in red. (**a**) Sr0; (**b**) Sr2; and (**c**) Sr5 (scale bars: 100 µm); and analysis of cell morphology assessed by SEM: (**d**) MG63 grown for three days on Sr2 surface (scale bar: 50 µm) with a higher power view showing details of the cell plasma extensions (**e**) (scale bar: 20 µm).

The incorporation of both strontium and alginate did not induce cytotoxicity, leading to a higher percentage of viable cells. The intracellular esterase activity and plasma membrane integrity were preserved. Only a few dead cells (stained in red) were observed (Figure 8a–c). Analysis of cell morphology carried out with SEM showed nearly complete coverage of Sr2 cement by well-spread cells (Figure 8d) and cell plasma extensions were evident (Figure 8e).

### 2.4. In Vivo Pilot Experiment and Histological Analysis

**Figure 9:**
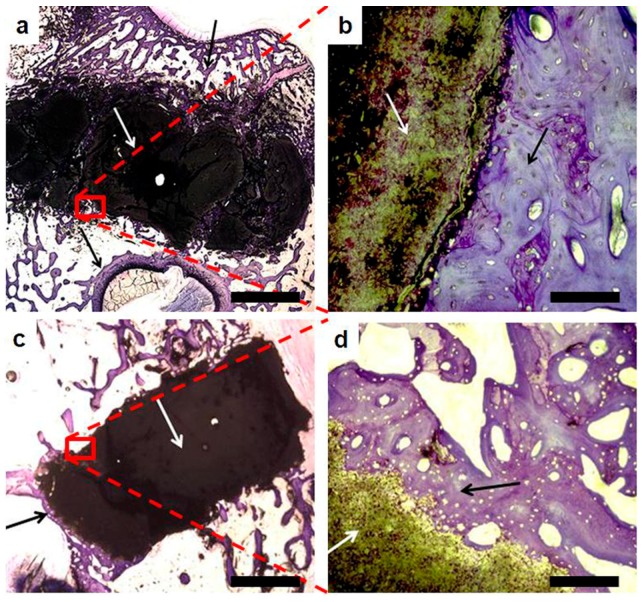
Optical microscopic view of explanted Sr2 (**a**,**b**) and KyphOs (**c**,**d**) at four weeks after implantation. Cements and surrounding bone are indicated with white and black arrows, respectively. Scale bars (**a**,**c** = 2 mm; **b**,**d** = 100 µm).

A pilot in vivo test was carried out to investigate the performance of Sr2 cement, selected for composition, setting, and the mechanical properties in accordance with the clinical need, in comparison with KyphOs FS™, Medtronic Spine LLC, Minneapolis, MN, USA, a polymer-free commercial CPC [8]. The histological analysis of the explants at four weeks after the injection in vivo evidenced an intimate contact between the bone cements and the surrounding bone, without inflammatory processes, fractures or infections (Figure 9). 

**Figure 10:**
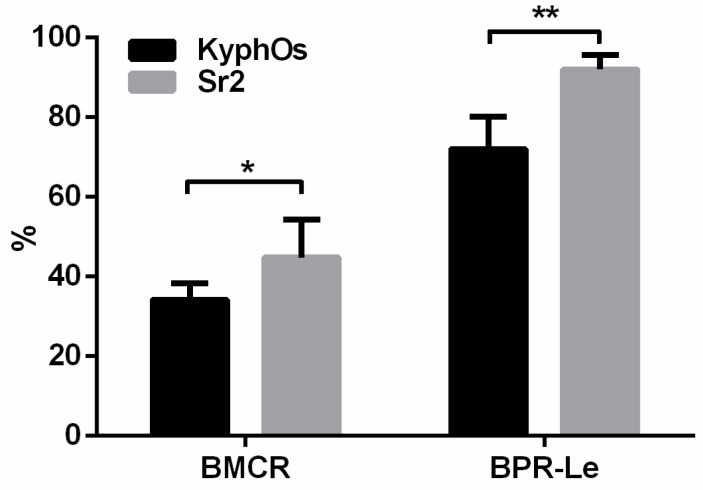
Histomorphometric data. The indicators BMCR and BPR-Le are explained in Table 4. * *p* < 0.5; ** *p* < 0.1.

The histomorphometric parameters bone-material contact rate (BMCR) and bone penetration rate length (BPR-Le) (see Table 4) were used to quantify bone-material interaction and new bone penetration, thus showing significantly enhanced formation of new bone around Sr2 cement, when compared with the control cement (Figure 10).

**Figure 11:**
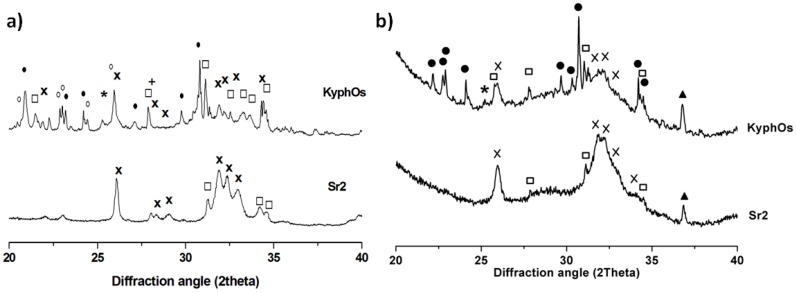
XRD patterns of Sr2 and KyphOs FS cement. (**a**) Cements hardened in vitro; and (**b**) explanted samples treated with both cements. Peaks legend: *α*TCP (•), *β*TCP (□), HA (×), SrCO_3_ (*), Mg_3_(PO_4_)_2_ (○), MgHPO_4_ (+), aluminum of the sample-holder support (▲).

Explanted tissues treated with Sr2 and control cement were subjected to XRD analysis to investigate the phase composition. Complete transformation of *α*-TCP into HA was detected in the sample treated with Sr2, so that only low-crystallinity HA and unreacted *β*TCP were detected. Additionally, the profile of the HA pattern exhibited a significant peak broadening, if compared with the HA phase obtained after immersion of Sr2 for three days in Hanks’ balanced salt solution at 37 °C, thus suggesting a decrease in the crystal order possibly due to the long term permanence of the cement in vivo (Figure 11). In particular, the domain size along (300) direction (i.e., the *ab* plane) was reduced from 19.9 ± 0.7 to 9.6 ± 1.8 nm, whereas along the (002) direction (i.e., the *c* axis) it decreased from 62.9 ± 1.6 to 34.3 ± 2.2 nm. The explanted tissue treated with KyphOs showed the presence of a low-crystallinity HA phase, but was associated with a significant amount of residual precursor phases, including *α*TCP, thus indicating that the transformation process was not complete after one month in vivo. 

**Figure 12:**
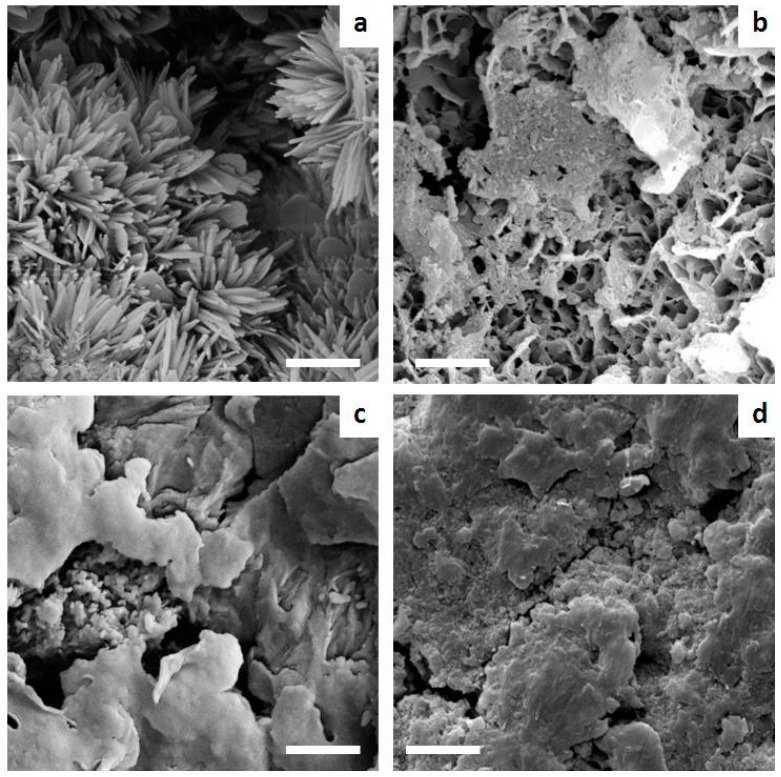
SEM images comparing the microstructure of Sr2 and the control cement after setting in vitro and in vivo. (**a**) Sr2 in vitro; (**b**) Sr2 in vivo; (**c**) KyphOs in vitro; and (**d**) KyphOs in vivo. Scale bars = 1 µm.

Furthermore, SEM analysis of the explants highlighted marked difference in the cement microstructure after setting in vitro and in vivo, as Sr2 still exhibited after implantation a diffuse microporosity after one month in vivo, contrary to the control (Figure 12).

## 3. Discussion

In the present work, Sr-substituted *α*TCP phases were synthesized as a unique inorganic precursor for the development of apatitic bone cements. For the first time, Sr-substituted apatitic cements were obtained by incorporating sodium alginate and tested both in vitro and in a small in vivo pilot study. The cements were optimized to fulfill specific requirements for clinical applications in terms of hardening times and mechanical strength [42,43].

The precursor synthesis, based on solid state reactions followed by fast quenching, generated Sr-doped *α*TCP phases with limited amounts of *β*TCP polymorph, slightly increasing with the strontium content, as previously reported [3]. This results are in compliance with the larger ionic radius of strontium compared with calcium (Sr^2+^ ionic radius = 0.113 nm; Ca^2+^ ionic radius = 0.099 nm) and in good quantitative agreement with the data provided in previous works [38,39,40,41]. In this respect, the detection of Sr amounts very close to the nominal composition of the starting mixture (Table 1), associated with the absence of secondary phases other than TCP (Figure 1) and the simultaneous increase of the lattice parameters (Figure 2), confirmed the incorporation of strontium into the TCP crystal lattices and in the final HA phase obtained upon cement setting. 

The achievement of complete injectability, associated with adequate setting times, is reported to be one of the most critical issues of CPCs [8]; in this respect, the addition of polymeric additives into CPCs has been widely investigated in previous research to enhance the rheological properties, but the precise control of their effect still remains challenging [44]. 

In our cements, characterized by ionic substitutions with strontium and the presence of alginate, a significant retard in both setting times (Table 3), and the transformation of TCP phases into apatite (Figure 4) were detected. However, this effect can be mainly ascribed to the presence of strontium, as also hypothesized in previous works [3,20,45], whereas the effect of alginate appeared as negligible. 

As a general rule, comparative analyses among the various cements reported in literature are made quite difficult by the variety of different, and often incomparable, preparation routes [20], thus often leading to contradicting results. 

It was reported that the incorporation of biopolymers can improve the functional properties of CPCs [44]. In this respect, despite previous research reporting the addition of alginate as deleterious for the mechanical performance of *α*TCP-based CPCs formulations [46], in the present work the use of alginate significantly improved both injectability and cohesion (Figure 3), leading also to significantly higher mechanical strength when compared with alginate-free cements (Figure 6). The effect of strontium on the mechanical strength appeared as negligible in alginate-free cements up to Sr2, while Sr5 exhibited the lowest values; a similar trend, but with greater statistical difference, was also detected among the alginate-containing cements, with Sr2 exhibiting the highest compressive strength (Figure 7), even comparable to previously-reported CPCs reinforced with fibers [15].

The reinforcement mechanism provided by alginate could be due to a crack bridging effect, similarly to what proposed by [47] in the case of cellulose-containing CPCs. 

On the other hand, a previous study reported a decrease of the mechanical strength of Sr-CPCs above a certain threshold of strontium [48], possibly due to the higher occurrence of crystal lattice distortion related to strontium’s larger ionic radius; in our study, we observed a similar effect for Sr/(Sr + Ca) above 2 mol %.

For the first time the biological effect of strontium doping and alginate into an apatitic cement was tested with MG63 cells, resulting in excellent proliferation and spreading of the seeded cells (Figure 8). This finding confirms previous studies reporting beneficial effects of strontium on cell proliferation in vitro [20], while the absence of cytotoxic effects related to alginate was assessed, supporting its use in the development of biomaterials for bone regeneration. 

On the basis of adequate cement composition and setting time for clinical applications [42,43], the Sr2 cement was selected to be mechanically and in vivo tested in comparison with KyphOs, a commercial strontium-containing apatitic cement [8] as control. After one month in vivo the Sr2 cement showed significantly higher bone-material contact and bone penetration (Figure 9 and Figure 10), perhaps due to the higher porosity. It might be also hypothesized that the presence of alginate favored the penetration of bone tissue into the cement by progressive bio-erosion in vivo. Comparative in vivo tests should be performed to support this hypothesis; however, in the present work we found that alginate-free pastes did not reach acceptable rheological and mechanical properties, as well as adequate setting times when injected in liquid media, so as to be considered as not interesting for real applications.

The XRD analysis of the explanted tissues reported that the composition of the control cement did not change significantly after one month in vivo, with only a partial conversion of the precursors into hydroxyapatite. Conversely, Sr2 exhibited complete transformation into hydroxyapatite in vivo, thus leading to the establishment of a completely biomimetic composition (Figure 11). In Sr2, the analysis of the HA peak profile evidenced a marked reduction of the crystal domain size. In this respect, it can be hypothesized that Sr2 underwent also a degradation process following the new bone formation and penetration.

The SEM analysis of the explants reported the maintenance of an extensive microporosity in Sr2 even after one month in vivo, while a more packed structure was exhibited by the control cement, that might have limited bone penetration into the cement mass (Figure 12). KyphOs exhibited significantly higher compressive strength but also lower overall porosity (Figure 7). Porosity substantially influences both the biological activity and the mechanical properties of biomaterials, therefore their optimization is a key aspect in the development of a bone cement. The microporosity of CPCs, which usually varies between 30% and 55%, is significantly dependent on the L/P ratio: the higher the L/P ratio, the higher the microporosity [15]. Therefore, to optimize these two properties, L/P ratio was maintained at the lowest value enabling complete injection and adequate cohesion. The final L/P of Sr2 resulted slightly higher than the control cement, i.e., 0.48 and 0.43, respectively; this yielded a much higher porosity associated with nanosized elongated crystals, as typically occurs in apatitic CPCs [3,49,50], which might have favored osteointegration. 

The bioactivity and effective osteointegration of a bone scaffold are key aspects to trigger and sustain bone regeneration in critical size defects [51]. In this respect, the approach presented in this work enabled the preparation of Sr-substituted HA cements with controlled and biomimetic composition, with defined strontium content replacing calcium in the lattice of HA, associated to excellent injectability, cohesion, and stable compressive strength enabling mechanical loading. The new cement maintained also high bioactivity and porosity in vivo, making these new pastes interesting for future applicative purposes. 

Although other cement formulations (e.g., PMMA-based cements) enable the early-stage mobilization of the patient after surgery due to high mechanical strength, their intrinsic bioinertness prevent effective bone regeneration. In this respect, the implantation of bioactive apatitic pastes able to completely transform into apatite while maintaining a microporous structure can be elective for bone regeneration, due to the extensive colonization by new bone that can also improve the mechanical performance of the implant over time, as already evidenced in previous studies [52,53]. This is particularly relevant in the case of young and still physically active patients, for whom effective bone regeneration is of paramount importance to restore the physiological bone functionality. 

## 4. Experimental Section 

### 4.1. Preparation of the Inorganic Precursors 

Sr-substituted *α*TCP powders with different strontium content (i.e., Sr/(Ca + Sr) = 0, 2, 5 mol %, henceforth coded as Sr0, Sr2, Sr5, respectively) were synthesized by solid state reaction of stoichiometric amounts of calcium carbonate (CaCO_3_, Sigma Aldrich, St. Louis, MO, USA), dicalcium phosphate dibasic anhydrous (CaHPO_4_, Sigma Aldrich) and strontium carbonate (SrCO_3_, Sigma Aldrich), following the reaction:

$$xSrCO_{3}+\left(1-x\right)CaCO_{3}+2CaHPO_{4}\to Ca_{3-x}Sr_{x}{\left(PO_{4}\right)}_{2}+\;H_{2}O+\;CO_{2}$$

The powders were dry mixed for 30 min, then pressed to form pellets and finally treated at 1400 °C for 1 h in a platinum crucible. The pellets were removed from the hot furnace and rapidly crushed into small pieces to stabilize the *α*TCP phase against the re-crystallization of *β*TCP [5]. Then, the as-obtained product was sieved below 150 µm and milled by planetary mono mill (Pulverisette 6 classic line, Fritsch, Germany). In detail, the powder was dispersed in pure ethanol and milled for 50 min at 400 rpm using a zirconia jar with 5 mm diameter grinding media (ball-to-powder weight ratio = 2). 

Aqueous solutions of disodium hydrogen phosphate dehydrate, 5 wt % (Na_2_HPO_4_·2H_2_O, Fluka), and sodium alginate, 2 wt % (Alginic Acid Sodium Salt from Brown Algae, Sigma Aldrich) were prepared.

The solid and liquid components were sterilized with *γ* irradiation (25 kGy) and autoclave (121 °C for 20 min), respectively, and mixed with the powders according to a liquid-*to*-powder (L/P) ratio equal to 0.48. The mixing procedure of the cement components and the extrusion of the cement were performed by commercial equipment (P-system, Medmix, Rotkreuz, Switzerland). 

Polymer-free formulations were obtained without adding sodium alginate into the solution.

The control cement (KyphOs FS™, Medtronic Spine LLC, Minneapolis, MN, USA), was prepared by mixing the powder and the liquid [8] with liquid-on-powder ratio equal to 0.43, according to the procedure included in the commercial package. 

### 4.2. Characterization of the Pastes and Hardened Cements

#### 4.2.1. Composition and Crystallographics

The elemental composition was evaluated by inductively-coupled plasma optical emission spectroscopy (5100 ICP-OES, Agilent Technologies, Santa Clara, CA, USA). The cement precursors were analyzed by dissolution of 20 mg of powders into 2 mL of nitric acid (HNO_3_, Sigma Aldrich), followed by dilution to 100 mL with bi-distilled water; three specimens were tested for each cement formulation.

The XRD patterns were acquired in the 2*θ* range 10°–80°, with a scan step of 0.02°, counting 0.5 s (D8 Advance, Bruker AXS equipped with a LINXEYE detector, Karlsruhe, Germany). Upon subtraction of the instrumental broadening, the collected XRD patterns were evaluated by Rietveld method (TOPAS 5 software, Bruker AXS, Karlsruhe, Germany) to simultaneously refine the scale factor, peak position, and peak broadening, thus obtaining the content of crystalline phases present, the lattice parameters and domain sizes. The Rietveld analysis was carried out by using the crystal models of Sr-free *α*TCP [54], *β*TCP [55] and HA [56] phases. The hardened cements were analyzed after three days of soaking in Hanks’ balanced salt solution (H6648, Sigma Aldrich) at 37 °C.

#### 4.2.2. Injectability and Setting Times 

The cement injectability was qualitatively evaluated by measuring the amount of cement in the syringe after extrusion [33]. The initial and final setting times of the cements (indicated as T_in_ and T_fin_, respectively) were evaluated by Gillmore apparatus, according to ASTM C266-99, testing five specimens for each composition, upon immersion in Hanks’ balanced salt solution at 37 °C. On the basis of setting times appropriate for the clinical practice, we considered an initial setting time ≤15 min as a threshold.

#### 4.2.3. Morphological Characterization

The morphology of the cements was observed by scanning electron microscopy (SEM: FEI, Quanta 200, Hillsboro, OR, USA) after immersion in Hanks’ balanced salt solution at 37 °C for 3 days. The porosity percentage was evaluated on hardened cylindrical specimens, as P = 1 *− ρ*/*ρ*_0_*,* where *ρ* is the cement density calculated as weight-on-volume ratio and *ρ_0_* is the theoretical density of the hydroxyapatite phase (3.16 g/cm^3^).

#### 4.2.4. Mechanical Evaluation

The compressive strength and Young’s modulus of cements were evaluated by testing cylindrical specimens (n. 12 samples; diameter = 8 mm; height = 17 mm) obtained after hardening in teflon moulds for 30 min and then immersed in Hanks’ balanced salt solution at 37 °C for three days. The tests were performed in displacement control at 1 mm/min by a universal testing machine (MTS Insight 5, Eden Prairie, MN, USA). The planarity of the endplates was obtained by using an automatic surface grinder (VAM Rettificatrici, Cuggiono, Italy). 

Long term evaluation of a selected cement was carried out in agreement with ISO 9917 by testing cylindrical specimens (diameter = 8 mm; height = 17 mm) up to 30 days of soaking in Hanks’ balanced salt solution [57] at 37 °C (n. 12 samples were tested).

The Young’s modulus was approximated by calculating the slope of the linear, elastic portion of the stress–strain curves until the failure stress of the specimen. The statistical analysis of data was made by a two-way analysis of variance (ANOVA) followed by Sidak’s multiple comparisons test (GraphPad 6.0, GraphPad Software, La Jolla, CA, USA). 

#### 4.2.5. Cell Culture

The cytocompatibility of alginate-containing cements was investigated by direct cell culture with human osteoblast-like cell line (MG63 cell line) purchased from Lonza (Basel, Switzerland). The cells were cultured in Dulbecco Modified Eagle’s (DMEM)/F12 Medium (Gibco, Waltham, MA, USA), containing penicillin-streptomycin (100 U/mL–100 µg/mL) supplemented with 10% fetal bovine serum (FBS) and kept at 37 °C in an atmosphere of 5% CO_2_. For the experiments, cells were plated at 1.5 × 10^4^/cm^2^ and cultured for up to three days. Cylindrical cement specimens (diameter = 10 mm and height = 2 mm) were sterilized with three washes in ethanol 70% for 20 min followed by three washes in 1X PBS for 10 min each. Samples were then air dried and sterilized by UV irradiation for 30 min per side under a laminar flow hood and preconditioned for three days in cell culture medium supplemented with FBS and penicillin-streptomycin.

Then, samples were placed one per well in a 24 well plate and a drop of 50 µL containing the cell suspension was seeded on the center of the upper discs surface allowing the cell attachment for 30 min in the incubator, before adding 1.5 mL of cell culture medium into each well. 

All cell-handling procedures were performed in a sterile laminar flow hood. All cell culture incubation steps were performed at 37 °C with 5% CO_2_ and controlled humidity.

#### 4.2.6. Cell Viability Assay

A live/dead assay kit (Invitrogen, Carlsbad, CA, USA) was performed according to the manufacturer’s instructions. Briefly, the samples were washed with 1X PBS for 5 min and incubated with calceinacetoxymethyl (Calcein AM) 2 µM plus ethidium homodimer-1 (EthD-1) 4 µM for 15 min at 37 °C in the dark, the samples were rinsed in 1X PBS [58]. Images were acquired by an inverted Nikon Ti-E fluorescence microscope (Nikon, Tokyo, Japan). One sample per cement was analyzed at day three.

#### 4.2.7. Cell Morphology Evaluation

The samples were washed with 0.1 M sodium cacodylate buffer pH 7.4 and fixed in 2.5% glutaraldehyde in 0.1 M sodium cacodylate buffer pH 7.4 for 2 h at 4 °C, washed in 0.1 M sodium cacodylate buffer pH 7.4 and dehydrated in a graded series of ethanol for 10 min each. After sputter-coating with gold, the samples were observed by scanning electron microscopy.

#### 4.2.8. In Vivo Pilot Experiment and Histological Analysis

The in vivo performance of a selected Sr-doped cement was assessed by implantation in three New Zealand White (NZW) male rabbits (weighing about 3 kg) for each cement. The tests were conducted with previous approval of the institutional Ethical Committee strictly following Italian (Law by Decree, 27 January 1992, No. 116), European (Directive 86/609/EEC) and International Laws and Regulations (ISO 10993-2-Animal Welfare Requirements). The cements were implanted according to a previously reported procedure [18,59,60]. Briefly, a blind tunnel (diameter = 4 mm; length = 8 mm) was aseptically created under fluoroscopic control by a surgical drill in the meta-epiphyseal distal zone of both femurs, then irrigated with a saline solution and filled by injection with the cements. All the procedures were performed under general anesthesia (ketamine 10 mg/kg + xylazine 0.3 mg/kg Intramuscular, isoflurane + oxygen + nitrogen protoxide with mask) with daily veterinary control. At four weeks after surgery, the animals were pharmacologically euthanized with intravenous administration of Tanax. The femurs were then harvested and the surrounding soft-tissue was removed. The presence of deformities or fractures and the bone-cement interface were investigated. For histological analysis, the harvested specimens were fixed in phosphate-buffered 4% paraformaldehyde and stored at 4 °C for 12 h until processing. Then, the specimens were embedded in Technovit 7200, according to the manufacturer’s protocol (Exakt, Norderstedt, Germany). By using a saw microtome (Leica Microsystems Srl, Milano, Italy), three consecutive central sections (30 ± 10 µm) were cut and stained with toluidine blue. The region of interest (ROI) was made by tracing over the material followed by a 100 pixel increase to include the bone-cement interface.

The microscopic examination was performed by an optical microscope (Carl Zeiss Axioscop 40 equipped with Axiocam ICC 3 Zeiss, Oberkochen, Germany) and Axiovision 4.8 software (Zeiss, Oberkochen, Germany). Standard terms and nomenclature for bone histomorphometric analysis were used, according to [61]. The statistical analysis of data, expressed as mean ± SEM (standard error of the mean), was made by an unpaired *t*-test (GraphPad software 6.0). 

## Figures and Tables

**Table 1:** ICP analysis of the as-obtained Sr-*α*TCP precursors and final cements.

Nominal Sr Concentration	Actual Composition		
Sr/(Ca + Sr) (mol %)	Ca/P (mol)	(Sr + Ca)/P (mol)	Sr/(Ca + Sr) (mol %)
0	1.51	1.51	0
2	1.44	1.46	1.8
5	1.40	1.46	4.9

**Table 2:** Linear dependence of lattice parameters and volume vs. Strontium content: *y = y*_0_ + *m*·*x_Sr_*. *y*_0_ refers to Sr-free composition, *x_sr_*: strontium content (%), *m:* extent of variation with the strontium content.

***α*TCP**	***y*_0_**	***m***	**R^2^**
*a*	12,849 Å	0.0074	0.988
*b*	27,340 Å	0.0151	0.988
*c*	15,217 Å	0.0029	0.987
Beta angle	126.30°	0.01616	0.963
Cell volume	4308 Å^3^	5.46	0.989
Cell volume [38]	4318 Å^3^	4.86	–
***β*TCP**	***y*_0_**	***m***	**R^2^**
*a*	10,414 Å	0.0047	0.93
*c*	37,341 Å	0.01281	0.993
Cell volume	3501 Å^3^	4.45	0.967
Cell volume [39]	3507 Å^3^	4.90	–
**HA**	***y*_0_**	***m***	**R^2^**
*a*	9.45717 Å	0.00274	0.997
*c*	6.87681 Å	0.00324	0.998
Cell volume	532.8 Å^3^	0.56	0.998
Cell volume [40]	528.0 Å^3^	0.85	–
Cell volume [41]	527.0 Å^3^	0.71	–

**Table 3:** Setting times of Sr-substituted formulations.

Setting Solution	Cement	T_in_ (min)	T_fin_ (min)
without Alginate	Sr0	10 ± 1	22 ± 3
	Sr2	13 ± 1	30 ± 3
	Sr5	23 ± 3	47 ± 3
with Alginate	Sr0	9 ± 2	17 ± 2
	Sr2	11 ± 2	26 ± 3
	Sr5	25 ± 3	51 ± 2

**Table 4:** Parameters observed and evaluated in histomorphometric analysis.

Parameter	Abbreviation	Formula
Material Perimeter	M-Pm	–
Bone Material Contact Length	BMC-Le	–
Bone Pores Length	BPo-Le	–
Material Diameter	M-Dm	–
Bone Material Contact Rate	BMCR	(BMC-Le/M-Pm) × 100
Bone Penetration Rate Length	BPR-Le	(BPo-Le/(M-Dm/2)) × 100
